# Systemic analysis of osteoblast-specific DNA methylation marks reveals novel epigenetic basis of osteoblast differentiation

**DOI:** 10.1016/j.bonr.2017.04.001

**Published:** 2017-04-03

**Authors:** Fangtang Yu, Hui Shen, Hong-Wen Deng

**Affiliations:** a Center for Bioinformatics and Genomics, Department of Global Biostatistics and Data Science, School of Public Health and Tropical Medicine, Tulane University, New Orleans, LA 70112, USA; b College of Life Sciences, Hunan Normal University, Changsha, Hunan 410081, China; c College of Life Sciences and Bioengineering, Beijing Jiaotong University, Beijing 100044, China

**Keywords:** Osteoblast, Methylation, Cell-specific, Differential methylation analysis, Transcription, Alternative splicing

## Abstract

DNA methylation is an important epigenetic modification that contributes to the lineage commitment and specific functions of different cell types. In this study, we compared ENCODE-generated genome-wide DNA methylation profiles of human osteoblast with 21 other types of human cells in order to identify osteoblast-specific methylation events. For most of the cell strains, data from two isogenic replicates were included, resulting in a total of 51 DNA methylation datasets. We identified 852 significant osteoblast-specific differentially methylated CpGs (DMCs) and 295 significant differentially methylated regions (DMRs). Significant DMCs/DMRs were not enriched in CpG islands (CGIs) and promoters, but more strongly enriched in CGI shores/shelves and in gene body and intergenic regions. The genes associated with significant DMRs were highly enriched in biological processes related to transcriptional regulation and critical for regulating bone metabolism and skeletal development under physiologic and pathologic conditions. By integrating the DMR data with the extensive gene expression and chromatin epigenomics data, we observed complex, context-dependent relationships between DNA methylation, chromatin states, and gene expression, suggesting diverse DNA methylation-mediated regulatory mechanisms. Our results also highlighted a number of novel osteoblast-relevant genes. For example, the integrated evidences from DMR analysis, histone modification and RNA-seq data strongly support that there is a novel isoform of *neurexin-2* (*NRXN2*) gene specifically expressed in osteoblast. *NRXN2* was known to function as a cell adhesion molecule in the vertebrate nervous system, but its functional role in bone is completely unknown and thus worth further investigation. In summary, we reported a comprehensive analysis of osteoblast-specific DNA methylation profiles and revealed novel insights into the epigenetic basis of osteoblast differentiation and activity.

## Highlights

• Identify and characterize osteoblast-specific methylation patterns across the genome
• Osteoblastic-specific methylation enriched in regulatory regions beyond the promoters
• Diverse, context-dependent DNA methylation-mediated regulatory mechanisms
• Revealed novel insights into the epigenetic basis of osteoblast differentiation

## 1 Introduction

DNA methylation of cytosine is a crucial epigenetic mechanism for transcriptional regulation and has profound impacts on embryonic development, genomic imprinting, X-chromosome inactivation, and the pathogenesis of various human disorders (Weber et al., 2007). Though the regulatory function of DNA methylation is generally thought to be associated with transcriptional repression when occurring in gene promoter regions and with transcriptional activation when occurring in gene bodies (Jones, 2012, Ball et al., 2009, Rauch et al., 2009), recent studies revealed a far more complicated relationship between DNA methylation and gene expression. Both negative and positive correlations between methylation and expression have been observed across all gene structural regions, and DNA methylation can also regulate alternative splicing through effects on RNA Pol II elongation (Jones, 2012, Shukla et al., 2011, Chandra et al., 2014, Ehrlich and Lacey, 2013, Liu et al., 2013, Deaton et al., 2011, Lee et al., 2014, Varley et al., 2013), indicating that DNA methylation can have diverse, chromatin context- and cell type-dependent regulatory functions on transcription.

With recent advance in high-throughput technology for DNA methylation analysis (Sun et al., 2015), a number of studies have demonstrated that DNA methylation profiles vary in diverse human tissues and cell types (Jones, 2012, Lokk et al., 2014, Yang et al., 2015), which contribute to the regulation of cell type-specific gene expression and determine the differentiation and specific function of different cell types (Futscher et al., 2002, de la Rica et al., 2013, Tsumagari et al., 2013). For example, Ziller et al. (2013) found that 21.8% of autosomal CpGs showed dynamic DNA methylation changes in a range of human cell and tissue types and these dynamic CpGs co-localized with gene regulatory elements, particularly enhancers and transcription-factor-binding sites, allowing identification of key lineage-specific regulators. In addition, Rica et al. (2013) identified hyper-/hypo-methylation changes in several thousand genes during in vitro monocyte-to-osteoclast differentiation, including all relevant osteoclast differentiation and function categories. DNA methylation has also been implicated in the regulation of differentiation and function of osteoblasts, the bone-forming cell with main function of mineralizing the bone matrix (Eslaminejad et al., 2013). For example, the promoter of *osteocalcin* gene, a gene solely expressed by osteoblasts, is highly methylated in cells not expressing osteocalcin, including the mesenchymal stem cells (MSCs) (Villagra et al., 2002). Interestingly, during in vitro MSC-to-osteoblast differentiation, as the osteocalcin gene becomes increasingly expressed, CpG methylation of the osteocalcin promoter is significantly reduced (Villagra et al., 2002). Similarly, reduced DNA methylation along with transcriptional upregulation were also observed for two additional osteogenic genes, namely, *alpha 1 type I collagen* (*COL1A1*) and *osteopontin* (Arnsdorf et al., 2010). In addition to hypomethylation mediated gene activation, hypermethylation induced silencing of specific genes were also crucial in osteoblast differentiation. For instance, Hsiao et al. (2010) found that *Trip10* (thyroid hormone receptor interactor 10), an adaptor protein involved in diverse cellular functions, shows significant alterations in promoter methylation and mRNA levels during lineage-specific induction of human bone marrow-derived MSCs. Remarkably, targeted induction of *Trip10* promoter methylation resulted in reduced *Trip10* expression and accelerated MSC differentiation towards osteogenic lineage at the expense of MSC-to-adipocyte differentiation. Taken together, these results strongly supported that DNA methylation plays a significant role in mediating cell-specific gene transcription and interfering with cell fate determination, including osteoblast differentiation.

In this study, we compared the genome-wide DNA methylation profiles between human osteoblasts and a wide range of other types of human cells in order to identify and characterize osteoblast-specific methylation patterns on a global scale. The purpose is to identify those genes and regulatory mechanisms underlying specific functions of osteoblasts. Our results revealed many osteoblastic hyper-/hypo-methylated CpGs across the genome. By integrating the DNA methylation patterns with transcriptomic and other epigenomic profiles, we further showed that these osteoblastic-specific methylation events were enriched in regulatory regions beyond the promoter areas and may influence gene expression and the use of alternative promoters in a cell-type specific manner. Collectively, these data may provide novel insight into the regulatory role of DNA methylation in osteoblast differentiation and functioning.

## 2 Results and discussion

### 2.1 Identification and characterization of osteoblast-specific DMCs/DMRs

We compared ENCODE-generated DNA methylation profiles of osteoblasts with those of 20 different types of non-transformed human cell strains plus Epstein-Barr virus-transformed lymphoblastoid cell lines (LCLs) (Supplementary Table 1). For most of the cell strains, DNA methylation data generated by reduced representation bisulfite sequencing (RRBS) from two isogenic replicates were included, resulting in a total of 51 DNA methylation datasets. The number of CpGs assessed per sample ranged from 960,300 to 1,489,630, including ~ 31.6–43.7% of CpGs with sequence coverage ≥ 10 × (Supplementary Table 1). We compared a total of 182,518 CpGs with coverage ≥ 10 × across all 51 samples and identified 852 significant differentially methylated CpGs (DMCs) with stringent criteria (q < 0.01, difference in methylation ≥ 50%), which were distributed across the entire genome (Supplementary Fig. 1). Hierarchical clustering analysis using the significant DMCs correctly grouped cells from similar tissues and of similar biological functions (Supplementary Fig. 2). Interestingly, we observed high similarity of the DNA methylation patterns between osteoblast and skeletal muscle myoblast. This is not completely unexpected, because both osteoblast and myoblast are mesodermal descendent of the bone-marrow mesenchymal stem cells (BMSCs) (Gimble et al., 2008). Moreover, it has been shown that myoblastic cells can be differentiated into osteoblastic cells (Tanaka et al., 2012), and the muscle-derived MSCs were more effective in differentiation into osteoblastic cells than BMSCs (Glass et al., 2011). In fact, a high similarity of chromatin states between osteoblast and skeletal muscle myoblast has also been observed in the NIH Roadmap Epigenomics project (C. Roadmap Epigenomics et al., 2015).

**Fig. 1:**
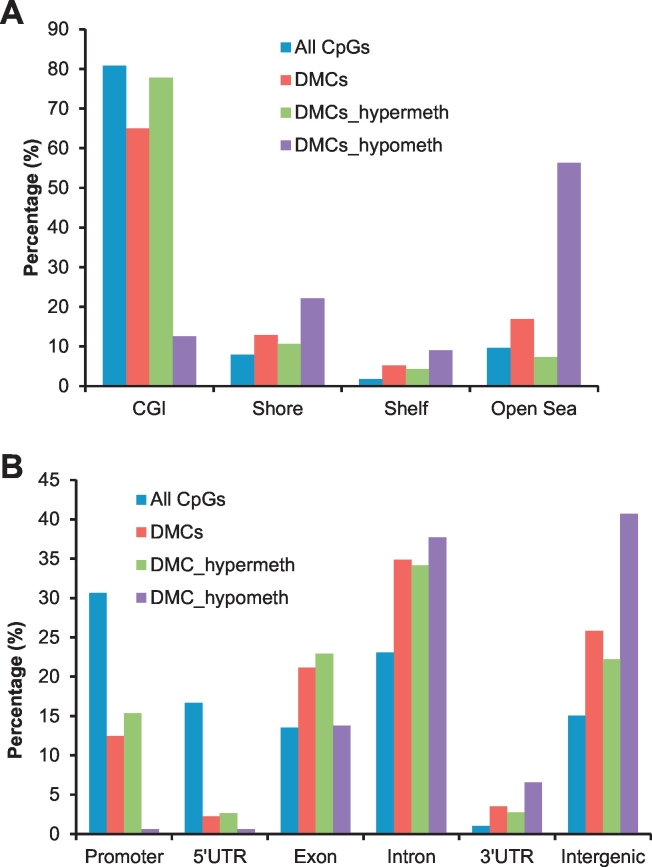
Distribution of all 182,518 tested CpGs and 852 significant osteoblast-specific DMCs, including 685 hypermethylated and 167 hypomethylated DMCs, across (A) different regions related to CGIs and (B) different genic regions.

Of the total 852 DMCs, 685 (80.40%) were hypermethylated and 167 (19.60%) were hypomethylated, in osteoblasts vs. other cell types. While the majority of the DMCs was mapped to CpG islands (CGIs), DMCs were more strongly enriched in non-CGI regions, including CGI shore (p = 5.73 × 10^− 7^, Fisher's exact test), CGI shelf (p = 7.54 × 10^− 11^) and open sea (p = 4.54 × 10^− 11^), when taking into account of the number of CpGs tested in each CpG annotation class (Fig. 1A). We observed a marked difference in the distributions with respect to CGIs between hyper- and hypo-methylated DMCs (Fig. 1A, p = 1.34 × 10^− 64^), with the over majority (78%) of hypermethylated DMCs associated with CGIs, in contrast to hypomethylated DMCs, which were mainly mapped to open sea (~ 56%) and relatively infrequent in CGIs (~ 13%). Interestingly, the enrichment of cell lineage-/tissue-specific DNA methylation events in non-CGI regions but depletion in CGIs have also been observed by others (Lokk et al., 2014, Yang et al., 2015, Byun et al., 2009, Slieker et al., 2013), highlighting the importance of exploring the functional significance of non-CGI methylation.

We considered the location of DMCs across different parts of individual genes. We observed a significant depletion of DMCs in 5′-untranslated regions (UTRs) (p = 4.2 × 510^− 34^) and promoters (p = 7.87 × 10^− 21^) but a significant enrichment of DMCs in exons (p = 1.64 × 10^− 07^), 3′UTR (p = 3.89 × 10^− 09^) as well as intergenic regions (p = 1.02 × 10^− 31^) (Fig. 1B), when comparing to the overall distribution of all the tested CpGs. These results are in line with previous findings that cell type-/tissue-specific epigenetic marks were more often found in gene body regions than in promoter areas (Lokk et al., 2014, Yang et al., 2015, Tsumagari et al., 2013, Ernst et al., 2011), suggesting that the intragenic DNA methylation may play significant roles in the differentiation of diverse cell/tissue types. There is also a significant difference in the genic distribution between hyper- and hypo-methylated DMCs (Fig. 1B, p = 5.89 × 10^− 11^). Although over 50% of both hyper- and hypo-methylated DMCs were mapped to gene body (introns and exons), the hypermethylated DMCs were observed much more frequently at promoters and 5-UTRs than hypomethylated DMCs, and the latter were more frequently associated with 3′-UTRs and intergenic regions.

While methylation at individual CpG sites may possibly be linked to gene expression regulation (Venza et al., 2012, Chen, 1983), methylation levels at neighboring CpGs are often highly correlated and methylation-mediated regulatory elements often extend across genomic regions (Davies et al., 2012, Baylin and Jones, 2011, Song et al., 2009). Therefore, we further conducted differentially methylated region (DMR) analysis based on the computed DMC data. In total, we identified 295 significant DMRs, including 247 (83.73%) hypermethylated and 48 (16.27%) hypomethylated DMRs in osteoblasts vs. other cell types. The full list of significant DMRs was provided in the Supplementary Table 2. The hypomethylated DMRs (mean size = 72 bp) were significantly longer (p = 0.008) but contained comparable numbers of individual DMCs (mean number of DMCs = 5, p = 0.10) than the hypermethylated DMRs (mean size = 61 bp), suggesting more spreading of demethylation than of de novo methylation. When mapping the 295 DMRs to different CGI regions, we found 197 (66.8%) DMRs were mapped to CGIs, 49 (16.6%) to CGI shores, 16 (5.4%) to CGI shelves, and 33 (11.2%) to open sea (Supplementary Fig. 3A). In addition, most of the DMRs were observed in gene bodies and intergenic regions rather than in promoters of RefSeq genes (Supplementary Fig. 3B). Similar to what we observed when comparing the CGI/genic distribution of hyper- vs. hypo-methylated DMCs, we observed a highly significant difference in the distribution of hyper- vs. hypo-methylated DMRs with respect to CGIs (Supplementary Fig. 3A, p = 3.22 × 10^− 25^) and RefSeq genes (Supplementary Fig. 3B, p = 3.22 × 10^− 25^). Specifically, the hypermethylated DMRs were much more frequently associated with CGIs and promoters than hypomethylated DMRs, and the latter were mainly in open sea and intergenic regions. This difference is also reflected by the distinct distribution of hyper- and hypo-methylated DMRs relative to the transcription start sites (TSSs) of associated genes (Supplementary Fig. 3C), with hypo-methylated DMRs showing a marked depletion within 5 kb of the TSSs but not for hyper-methylated DMRs.

To better understand the biological context of the osteoblast-specific DMRs, we examined the distribution of the DMRs with respect to the different chromatin states in osteoblasts, which were characterized by the NIH Roadmap Epigenomic Consortium (C. Roadmap Epigenomics et al., 2015). Overall, the distribution of hyper- and hypo-methylated DMRs in chromatin states were not significantly (p = 0.68) different. Hyper-/hypo-methylated DMRs were often associated with elements in weak transcription, repressed/quiescent chromatin states polycomb, but not with active/flanking TSS regions (Supplementary Fig. 3D), suggesting that these osteoblastic-specific DMRs mainly affect weakly/low-level transcribed elements rather than active promoters/TSS flanking regions.

**Fig. 2:**
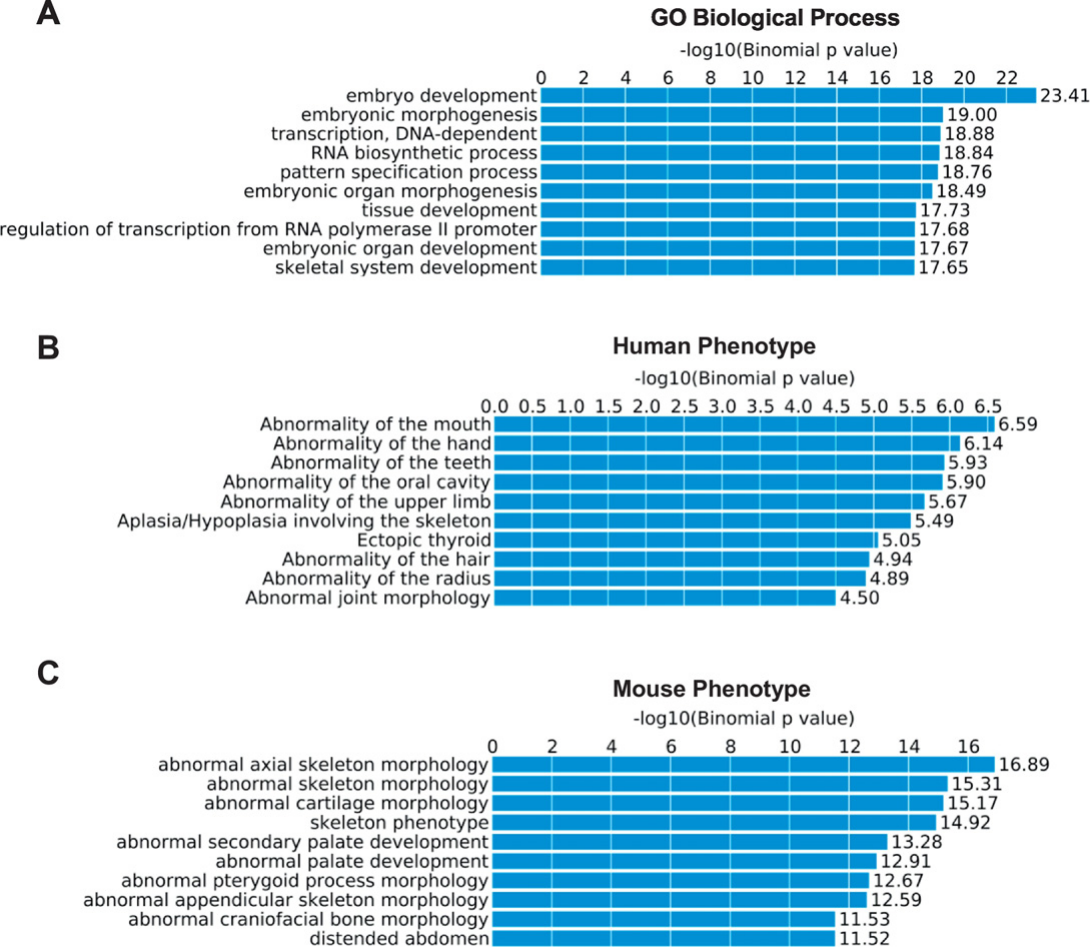
Top 10 results from functional annotation and GO enrichment analysis of osteoblast-specific hypermethylated DMRs by using the GREAT package (McLean et al., 2010).

To further explore the potential functional significance of the osteoblastic-specific DMRs, we next tested whether the nearby genes of DMRs were enriched for certain functional terms by using the GREAT program (McLean et al., 2010). The gene ontology (GO) enrichment analysis revealed that genes associated with the hypermethylated DMRs were highly enriched in a number of biological process terms that are relevant to embryo and skeletal development (Fig. 2A), such as embryonic development (p value = 3.91 × 10^− 24^, fold enrichment = 3.02) and skeletal system development (p value = 2.24 × 10^− 18^, fold enrichment = 3.86). Remarkably, the top 10 mouse and human phenotypes that were most significantly enriched for genes associated with the hypermethylated DMRs were almost all related to skeletal abnormalities (Fig. 2B–C), such as abnormal axial skeleton morphology (p-value = 1.03 × 10^− 18^, fold enrichment = 2.78), abnormal cartilage morphology (p-value = 1.70 × 10^− 17^, fold enrichment = 3.88), and abnormality of the mouth/hand/teeth (p-value = 2.58 × 10^− 7^–1.18 × 10^− 6^, fold enrichment = 2.07–2.52). In addition, by integrating with results from a large meta-analysis of genome-wide association studies (GWASs) for osteoporosis risk (Estrada et al., 2012), we demonstrated significant enrichment of osteoporosis-associated genes in both hyper- and hypo-methylated DMRs (p value = 2.00 × 10^− 4^ and 0.0187 respectively). Specifically, of the 178 genes annotated to hypermethylated DMRs, 6 genes (*ESR1*, *FOXL1*, *HOXC4*, *HOXC5*, *HOXC6*, and *WNT3*) showed significant genetic association with osteoporosis in the GWAS meta-analysis. Similarly, one (*PTPRN2*) of the 31 genes annotated to the hypomethylated DMRs are associated with osteoporosis risks. These are strongly contrasted with the background gene set, for which of the 11,329 genes annotated to all tested CpGs, only 77 genes were associated with osteoporosis. These results strongly suggested the identified DMRs and their associated genes may play functionally significant roles in bone metabolism and skeletal development in physiologic and pathologic conditions.

By integrative analysis of the DMR data with the extensive gene expression and chromatin epigenetics data in ENCODE (E.P. Consortium, 2012), we observed complex, context-dependent relationships between DNA methylation, chromatin states, and gene expression, which are illustrated below with some representative genes.

### 2.2 Hypermethylated DMRs at promoters/5′-end regions repress gene expression in osteoblasts: SIM2 and GLIS1

**Fig. 3:**
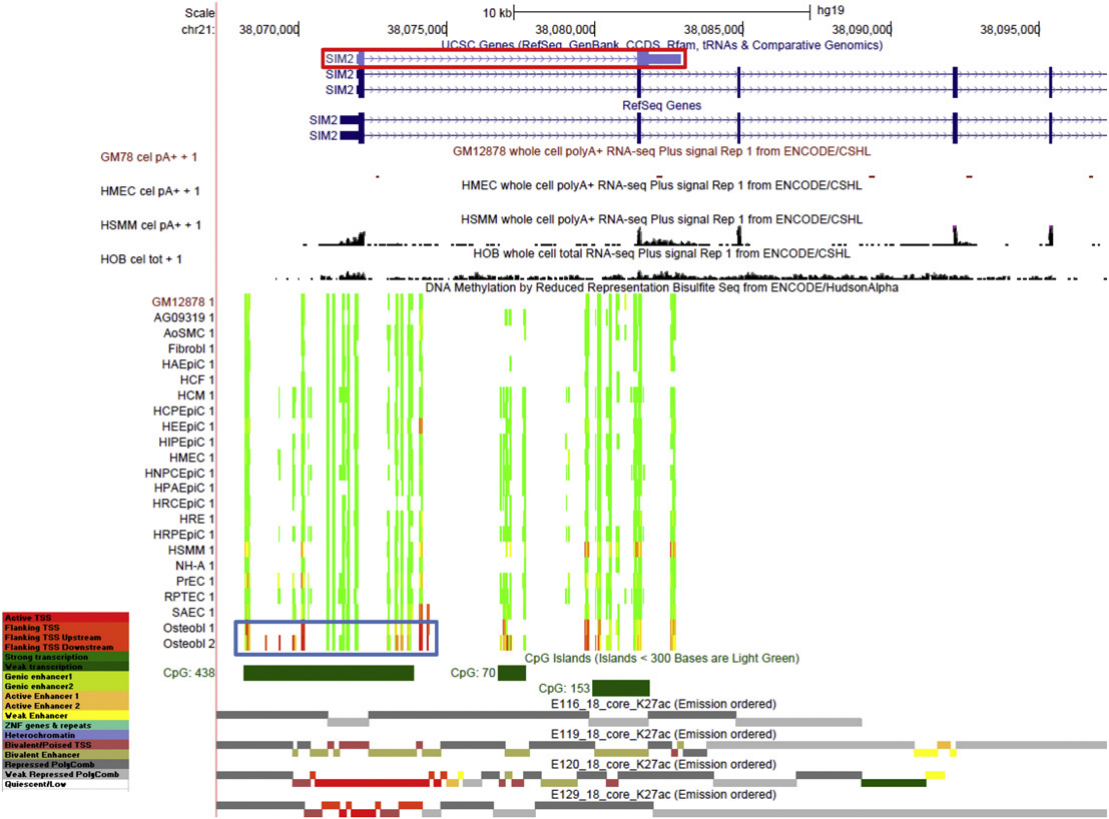
Osteoblastic hypermethylation at *SIM2* TSS surrounding region. The following profiles are shown using the UCSC Genome Browser (http://genome.ucsc.edu, version hg19) for the *SIM2* gene region: UCSC genes, RefSeq genes, RNA-seq data from ENCODE/Cold Spring Harbor, DNA methylation levels assessed by RRBS from ENCODE/HudsonAlpha, CpG islands, Chromatin state (18-state) annotation from NIH Roadmap Epigenome project. LCL (only GM12878 is shown, but the other LCL samples give similar results), HMEC, HSMM, and osteoblast (HOB) are the only studied cell types having both data in RNA-seq and histone modification tracks available from ENCODE. Methylation status is represented with an 11-color gradient for which red, yellow, and green represent that 100%, 50%, and 0% of molecules sequenced are methylated, respectively. Osteoblast-specific DMRs are indicated in the blue box. At this scale, individual differentially methylated CpG sites cannot be resolved from neighboring sites. The specific non-coding SIM2 isoform (Ensembl transcript ID: ENST00000460783.1) is marked in the red box. The 18 chromatin states are represented with the indicated colors, with E116 representing states for LCL (GM12878), E119 for HMEC, E120 for HSMM, and E129 for osteoblast. The detailed ChIP-seq data for various histone modification marks (H3K4me1, H3K4me3, H3K9me3, H3K27ac, H3K27me3, H3K36me3, H3K79me2, and H4K20me1) are presented in the Supplementary Fig. 3.

Promoters and 5′end regions are usually constitutively unmethylated, especially when they overlap with CGIs, even in genes with cell type-specific expression (Meissner et al., 2008). Nonetheless, there are notable exceptions as illustrated by gene *SIM2*. Specifically, we detected multiple osteoblast-specific hypermethylated DMRs (q-value = 8.57 × 10^− 19^–1.25 × 10^− 38^, DM% = 32.3–82.4%) at *SIM2* promoter and an immediate downstream region of its TSS (Fig. 3). These DMRs were all distributed within a large CGI.

Gene-repressive DNA hypermethylation in promoter regions normally localize to chromatin with repressive histone modification markers, such as H3K27me3 and H3K9me3 (Hagarman et al., 2013). However, this was not the case for the promoter region of *SIM2*. Instead, the promoter and 5′end region of *SIM2* display strong signal for active promoter (H3K4me3) and transcriptional elongation (H3K79me2) in osteoblast (Fig. 3 and Supplementary Fig. 4). Consistently, we also observed low but detectable levels of *SIM2* expression in osteoblast (RNA-seq track in Fig. 3). Similar active transcription chromatin states were also observed at this region in human skeletal muscle myoblasts (HSMM), but the expression levels of *SIM2* in HSMM were considerably higher. This suggested the osteoblast-specific DNA hypermethylation at this region imposes a repressive effect on the transcription of *SIM2*, even when separated from the typical promoter-inhibiting chromatin marks. We speculate that the co-existence of cell-specific DNA hypermethylation and active transcription/elongation chromatin marks, but lacking repressive histone modifications, at *SIM2* promoter/5′end regions allows a tight control of repressed but not completely abolished expression of this genes in osteoblasts. In contrast, the *SIM2* gene is completely silenced in lymphocyte B-cells (GM12891) and Human Mammary Epithelial Cells (HMEC), which are likely to be mediated by the strong and wide-spread repressive histone modification of H3K27me3 (Fig. 3 and Supplementary Fig. 4). *SIM2* gene encodes a transcription factor that is generally known as a mast regulator of neurogenesis. However, several studies indicated that *SIM2* also plays a critical role in the regulation of osteogenesis and skeletal development (Shamblott et al., 2002, Goshu et al., 2002, Kubo et al., 2009). Specifically, siRNA knockdown of *SIM2* in MSCs suppressed osteogenesis potential and delayed matrix calcification (Kubo et al., 2009), and *SIM2* knockout mice exhibited prominent craniofacial and vertebrae abnormalities (Shamblott et al., 2002, Goshu et al., 2002). On the other hand, over-expression of *SIM2* has been implicated in the pathogenesis of Down syndrome (Meng et al., 2006). Therefore, the temporal and spatial expression of *SIM2* may have to be tightly regulated to prevent pathological consequences in a cell type-specific manner, and DNA methylation may be a critical mechanism for this fine-tuning of expression. Interestingly, osteoblast-specific hypermethylated signals also extended to multiple CpGs further deep in the *SIM2* gene body (in introns 1–2 and exon 2), which precisely bound a potential non-coding *SIM2* isoform (Fig. 3). Therefore, hypermethylated DMRs may also regulate the *SIM2* isoform expression in osteoblast.

**Fig. 4:**
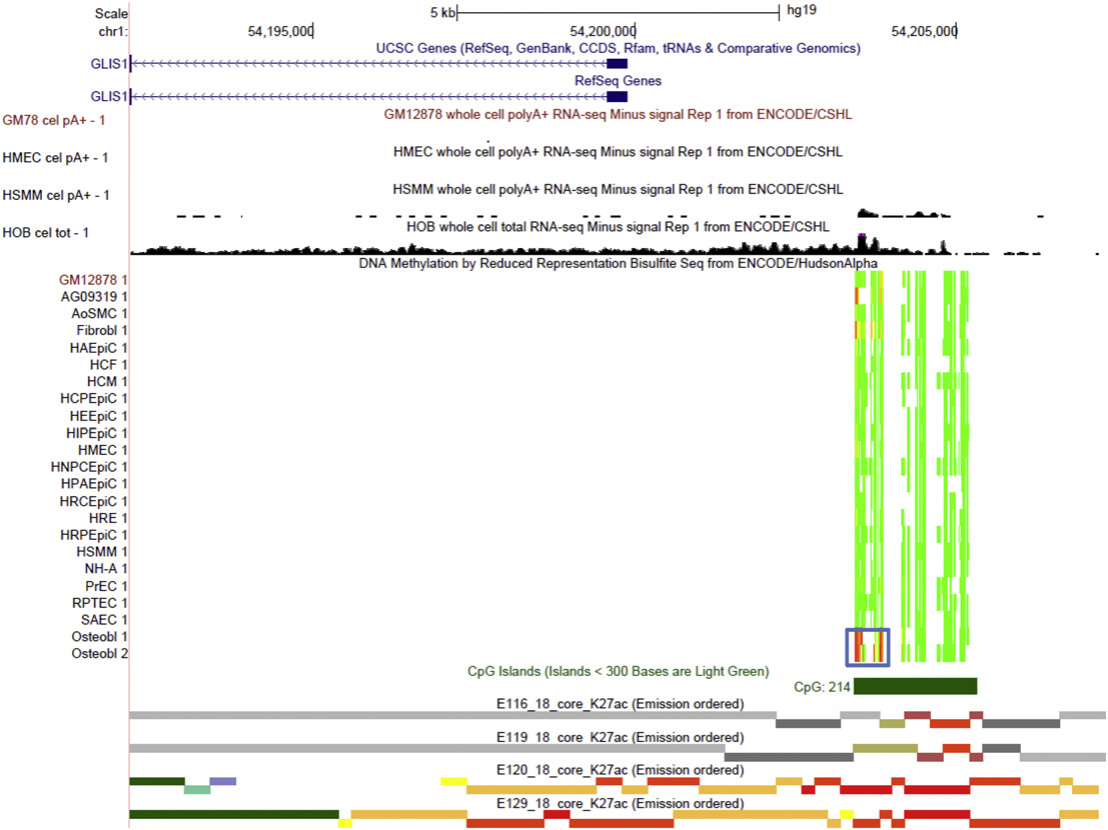
Osteoblastic hypermethylation at *GLIS1* TSS surrounding region. The same tracks as illustrated in Fig. 3 are shown using the UCSC Genome Browser (http://genome.ucsc.edu, version hg19). Osteoblast-specific DMRs are highlighted in the blue box.

Similar to *SIM2*, we also detected highly significant osteoblast-specific hypermethylated DMRs (q-value = 1.06 × 10^− 45^–1.08 × 10^− 49^, DM% = 64.8–69.5%) at the promoter of *GLIS1* gene, which overlap with a single CGI (Fig. 4). The RNA-seq and ExonArray data indicate *GLIS1* gene is preferentially expressed in osteoblasts and to a less extent, in HSMM, among the cells used for DMR detection (Fig. 4). Consistent with the gene expression data, histone modification marks indicate the existence of strong enhancers and active promoter (H3K4me3 and H3K27ac) at the *GLIS1* promoter regions specifically in osteoblast and HSMM, and poised promoter (H3K4me3 and H3K27me3 bivalent marks) in *GLIS1* non-expressing cells e.g., LBL, HMEC (Fig. 4 and Supplementary Fig. 5). Interestingly, the two osteoblast-specific hypermethylated DMRs precisely bound a segment exhibiting active promoter- and strong enhancer-like histone modification marks (H3K4me3 and H3K27ac) specifically in HSMM but barely detectable in osteoblasts (Supplementary Fig. 5). Therefore, these osteoblast-specific hypermethylated DMRs might repress a myogenic-specific promoter/enhancer in osteoblasts, allowing for precise regulation of *GLIS1* expression in a cell type-specific manner. *GLIS1* encodes for a GLI-related Kruppel-like zinc finger transcription factor and can effectively promote the reprogramming of somatic cells during induced pluripotent stem cells (iPSC) generation (Maekawa and Yamanaka, 2011, Maekawa et al., 2011). Importantly, GLIS1 is upregulated during the osteoblastic differentiation (Bustos-Valenzuela et al., 2011) and has been linked to coronary artery calcified plaque (Divers et al., 2013), which is closely related to osteoblastic differentiation and activity (Doherty et al., 2003). Together, these results suggest that *GLIS1* expression is likely to be tightly regulated in osteoblasts and cell type-specific DNA methylation may help to achieve this fine-tuning of expression.

### 2.3 Hypermethylation at alternative promoters contributes to cell type-specific isoform expression: MEST and NRXN2

**Fig. 5:**
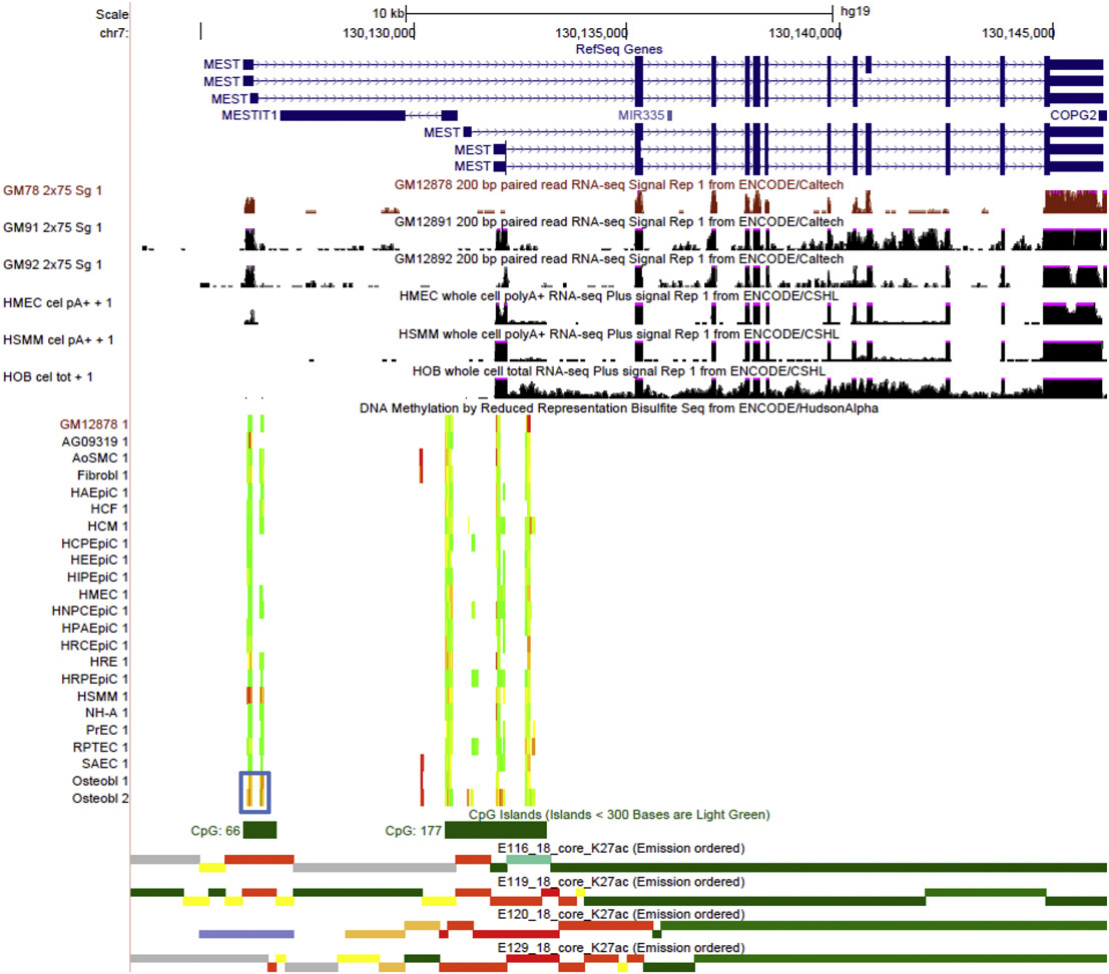
Osteoblastic hypermethylation at *MEST* gene region. The same tracks as illustrated in Fig. 3 are shown using the UCSC Genome Browser (http://genome.ucsc.edu, version hg19), with the additions of RNA-seq tracks for LCLs (GM12891 and GM12892) from ENCODE/Caltech. Osteoblast-specific DMR is highlighted in the blue box.

MEST (mesoderm specific transcript) is a member of the α/β hydrolase fold family and may play a role in development, including bone growth (Andrade et al., 2010). *MEST* gene has multiple, complicated mRNA isoforms, including 6 RefSeq annotates and at least 17 alternative mRNA variants identified by AceView program (Thierry-Mieg and Thierry-Mieg, 2006). The 6 RefSeq *MEST* annotates resulted from the usage of 4 alternative promoters/TSSs and 2 alternatively spliced exons (Fig. 5). Interestingly, we detected a significant osteoblast-associated hypermethylated DMR (q-value = 1.47 × 10^− 39^, DM% = 55.9%) overlapping one of the alternative promoters/TSSs that encode RefSeq transcript variants 2 and 5 (Fig. 5). CpGs within this DMR exhibited strong methylation in osteoblast and HSMM, but are largely unmethylated in other cell types, including LCL and HMEC (Fig. 5). The ENCODE RNA-seq data indicate that there are considerable transcription signals of *MEST* from this alternative TSS in LCL and HMEC, suggesting the expression of RefSeq transcript variants 2 and/or 5 in LCL and HMEC. In contrast, these two *MEST* alternative transcripts were barely detectable in osteoblast and HSMM (Fig. 5), for which *MEST* RefSeq transcript variants 1 and/or 4 are dominant. Consistently, there are strong active promoter/enhancer (H3K4me1 and H3K4me3) signals at this DMR-overlapped alternative TSS in LCL and HMEC, but not in osteoblast and HSMM (Fig. 5 and Supplementary Fig. 6). Interestingly, *MEST* is known to exhibit isoform-specific imprinting (Kosaki et al., 2000, Huntriss et al., 2013, Kamei et al., 2007) and the promoter switching may lead to loss of imprinting and aberrant expression of *MEST* gene, which has been linked to several types of cancers (Pedersen et al., 2002, Nestheide et al., 2013, Li et al., 2008). Particularly, aberrant expression of *MEST* gene has been detected in human osteoblast cell lines (hFOB1.19 cells) in a model of human osteosarcoma (Li et al., 2008). Taken together, DNA methylation may represent a critical epigenetic mechanism for regulation of alternative promoter usage at *MEST* gene in a cell-type specific manner, and dysregulation of this epigenetic mechanism may contribute to the pathogenesis of *MEST* loss-of-imprinting associated disorders.

**Fig. 6:**
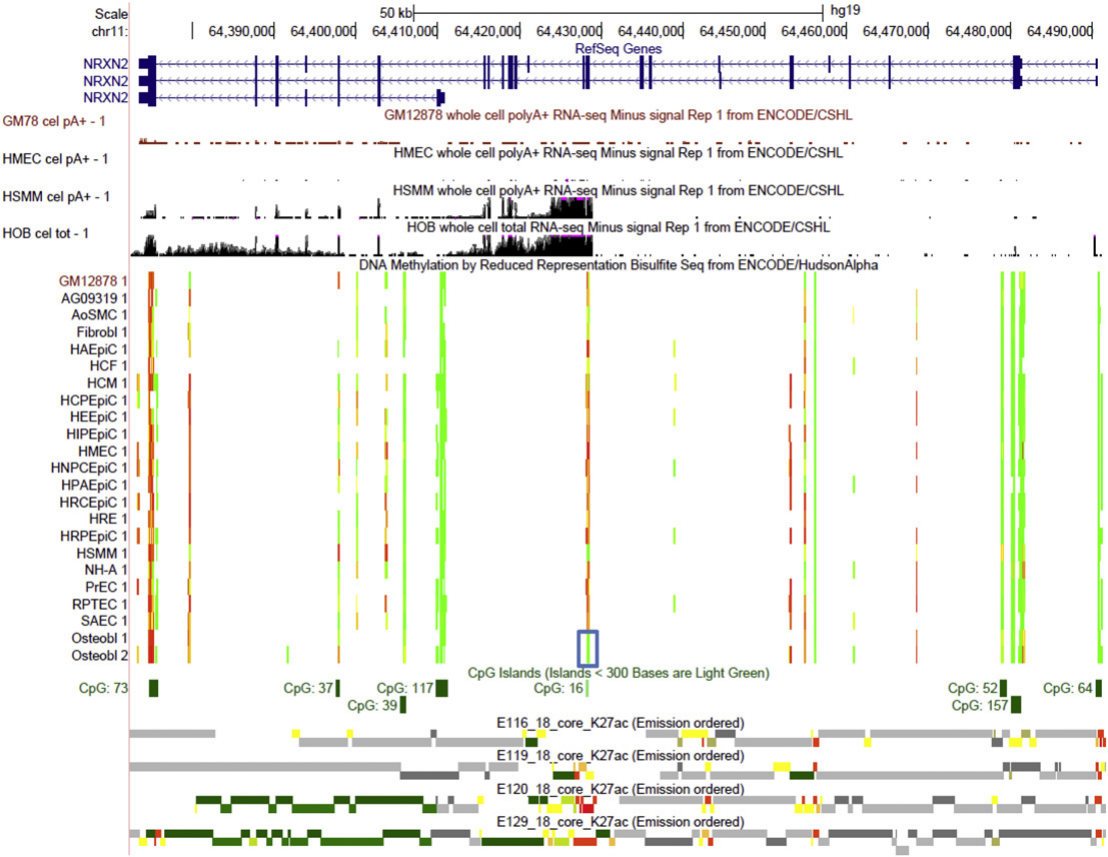
Osteoblastic hypomethylation at *NRXN2* gene region. The same tracks as illustrated in Fig. 3 are shown using the UCSC Genome Browser (http://genome.ucsc.edu, version hg19). Osteoblast-specific DMR is highlighted in the blue box.

Another potential connection between osteoblast-associated DMR and cell-type specific isoform expression was detected in *NRXN2* gene. *NRXN2* gene encodes a member of the neurexin gene family and has very complex transcription architecture. Though RefSeq annotates only 3 representative transcripts (Fig. 6), the alternative transcription of this genes is likely to be far more complicated, with 31 (annotated by NCBI *Homo sapiens* Annotation Release 107, Supplementary Fig. 7) and possibly thousands of alternative isoforms generated through the usage of multiple alternative promoters and extensive alternative splicing events (Tabuchi and Sudhof, 2002, Rowen et al., 2002). Specifically, we identified a significant, osteoblast-associated hypomethylated DMR (q-value = 1.58 × 10^− 51^, DM% = − 64.8%) spanning 8 CpGs in the exon 10 of the *NRXN2* RefSeq transcript variant alpha-1 (Fig. 6). Similar hypomethylation was also observed in HSMM, whereas almost all the other cell types exhibited hypermethylation in this region (Fig. 6). Interestingly, the ENCODE RNA-seq data indicate that a specific *NRXN2* isoform initiating from the immediate upstream of the exon 10 is highly expressed in osteoblast and HSMM, but not detected in LCL and HMEC (Fig. 6). Though the mRNA transcript of this specific isoform delineated by RNA-seq does not match any of the three RefSeq transcripts, it is consistent with the predicted *NRXN2* transcript variant X29 (XM_011545385.1) by the NCBI annotation (Supplementary Fig. 7), strongly supporting the authentic and predominant expression of this transcript variant in osteoblast and HSMM. Moreover, there were strong signals of active promoter-like (H3K4me3 and H3K27ac) and transcriptional activity-associated (H3K79me2) chromatin states around the DMR in osteoblast and HSMM, whereas these histone modification marks were depleted around the DMR in cell types that did not express this isoform, such as LCL and HMEC (Supplementary Fig. 7). These results strongly support that the intragenic DNA methylation may have a crucial role in regulating cell context-specific alternative promoters in gene bodies (Maunakea et al., 2010).

*NRXN2* is known as a cell surface protein involved in cell recognition and cell adhesion in the vertebrate nervous system. It plays an essential role in synapse function and its alterations have been linked to autistic spectrum disorder (Gauthier et al., 2011, Dachtler et al., 2014). The majority of *NRXN2* transcripts in the nervous system are produced from the upstream promoter and encode alpha-neurexin isoforms while a smaller number of transcripts are produced from the downstream promoter and encode beta-neurexin isoforms. The alpha-neurexins contain one epidermal growth factor-like (EGF-like) sequence and six laminin G domains, and have been shown to interact with neurexophilins. The beta-neurexins lack EGF-like sequences and contain only one laminin G domain, and bind to alpha-dystroglycan. The *NRXN2* variant X7 is also predicted to lack the EGF-like sequences but contain three laminin G domains. The functional roles of this NRXN2 variant in osteoblast warrant further exploration.

### 2.4 Hypomethylated DMRs at promoters contribute to active expression of primary osteoblastic genes: BGLAP

**Fig. 7:**
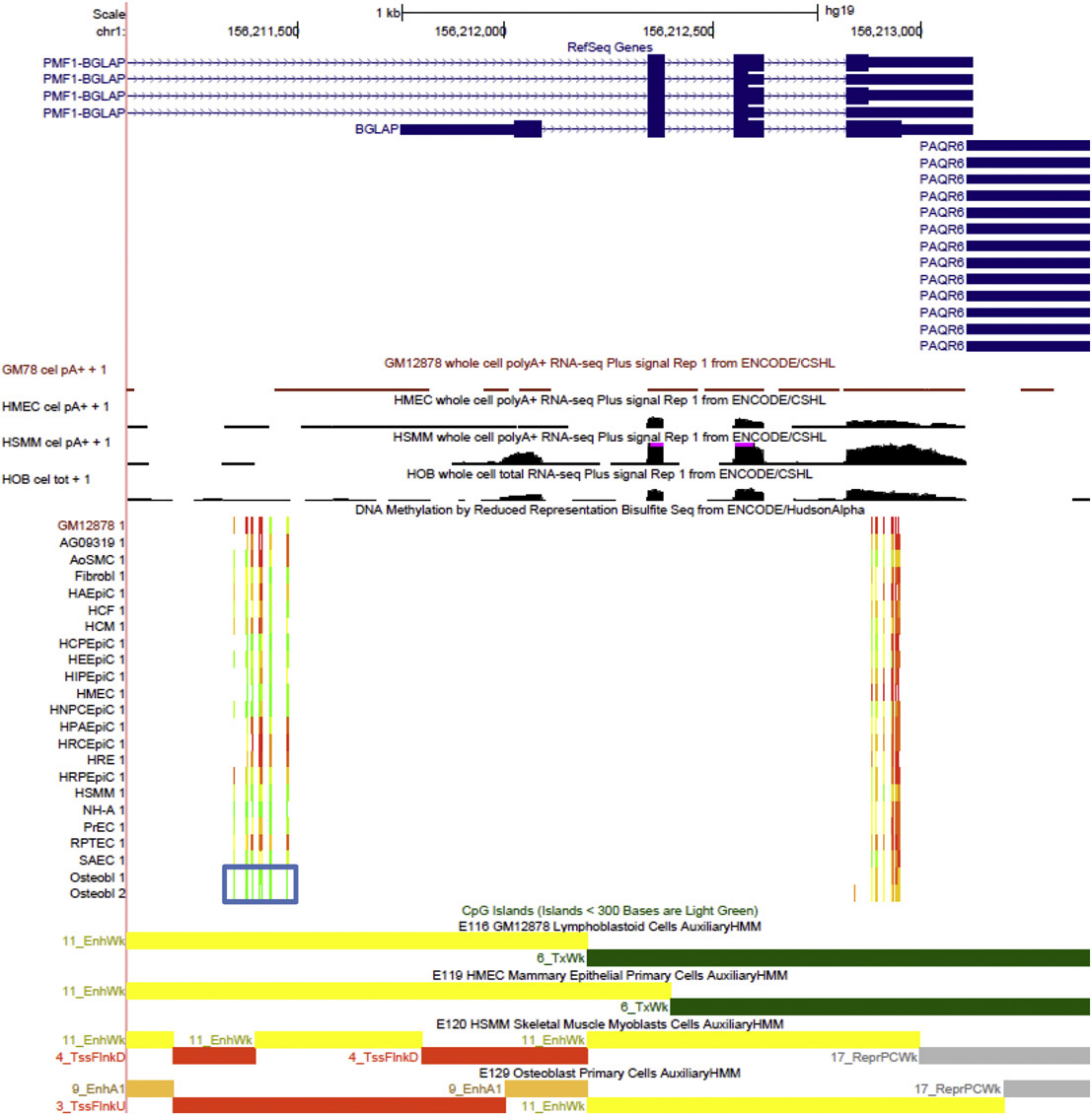
Osteoblastic hypomethylation at *BGLAP* gene region. The same tracks as illustrated in Fig. 3 are shown using the UCSC Genome Browser (http://genome.ucsc.edu, version hg19). Osteoblast-specific DMR is highlighted in the blue box.

We specifically examined the DNA methylation patterns around a number of genes that were known to play key roles in osteoblastic differentiation (Hojo et al., 2015, Kirkham and Cartmell, 2007, Cawthorn et al., 2012), including *Runx2* (*Cbfa1*), *Sp7* (*osterix*), *Dlx5*, *Msx2* (*HOX8*), *BGLAP* (*osteocalcin*), *COL1A1*, *MEF2C*, *BMPs* (*BMP-2, -4, -6, -7*, and *-9*), and *WNTs* (*WNT-6, -8, -10a* and *-10b*). We identified a highly significant osteoblast-specific hypomethylated DMRs (q-value = 7.15 × 10^− 45^, DM% = − 44.89%) at the promoter region of *BGLAP* gene (Fig. 7 and Supplementary Table 2). This significant DMR overlapped with a region showing strong active promoter-/enhancer-related chromatin states (Fig. 7) and histone modification marks (H3K4me1, H3K4me3, and H3K27ac) (Supplementary Fig. 8). These findings were consistent with the evident *BGLAP* expression in osteoblast (Fig. 7). In contrast, *BGLAP* promoter showed hypermethylation and/or weak enhancer-related chromatin marks in HMEC and LCL (Fig. 7 and Supplementary Fig. 8). Therefore, our findings provided direct evidence that promoter methylation may interactively work with other epigenomic mechanisms to regulate the cell-type specific expression of some key osteogenic genes. Interestingly, promoter hypomethylation and active transcription of *BGLAP* were also observed in HSMM (Fig. 7 and Supplementary Fig. 8). This again reflected the close connections between osteoblast and HSMM. In fact, a recent study has demonstrated that *BGLAP* expression in myofibers is necessary and sufficient to maintain muscle mass in mice (Mera et al., 2016).

In contrast, no significant DMRs were detected at other selected osteoblastic genes (including ± 5 kb upstream/downstream regions) (Supplementary Table 3). On one hand, this may reflect the inadequate coverage of genome-wide CpGs by RRBS, which was known to be biased towards regions rich in CpG sites (e.g., CGIs). For instance, no DNA methylation data were available for CpGs located within the ± 5 kb surrounding regions of *Runx2* gene from the ENCODE RRBS dataset. Future studies using more comprehensive DNA methylation techniques, such as whole-genome bisulfite sequencing, are needed to investigate the DNA methylation mediated regulations for these genes. On the other hand, the lack of significant DMRs in these selected osteoblastic genes may imply that various other mechanisms (e.g., histone modification) may regulate the expression of these genes independent of the effects of DNA methylation (Weber et al., 2007, Jones, 2012).

## 3 Summary

In this study, we identified and characterized human osteoblast-specific DNA methylation profiles by comparing the genome-wide DNA methylation profiles between human osteoblasts and 21 other types of human cells and by integrating the DNA methylation patterns with transcriptomic and other epigenomic profiles. This study has a few notable limitations. First, most of the analyzed cell types only have two isogenic replicates and thus the potential inter-individual variability of DNA methylation patterns within each cell type has not be taken into account. In addition, all the epigenomic and expression data were generated from cells expanded in vitro, which may exhibit distorted profiles from their in vivo status (Caliskan et al., 2011, Saferali et al., 2010). Despite these limitations, several evidences provided strong support for the general reliability of our findings. For instance, the identified osteoblastic-specific DNA methylation sites were distributed across different genomic regions in a pattern that was largely in agreement with the patterns previously observed by other tissue-/cell-type specific DNA methylation profiling studies. More importantly, the identified osteoblastic-specific DMRs were significantly enriched for genes that are critical for bone metabolism and skeletal development in physiologic and pathologic conditions, providing compelling evidence that DNA methylation may regulate transcription including cell-type specific isoform expression of many genes that are important for osteoblast differentiation and activities. Our results provided a framework for development of more specific hypotheses concerning epigenetic regulation of osteogenesis and highlighted several interesting targets for further evaluation. Future studies with multiple biological replicates and in vitro as well as in vivo functional assays are required to further replicate our findings and elucidate the molecular mechanisms underlying the DMR-mediated regulation of osteoblast differentiation and function, particularly for the numerous DMRs found in gene body areas.

## 4 Materials and methods

### 4.1 Samples and DNA methylation profiling

Genome-wide DNA methylation profiles of osteoblast and 20 additional different types of non-transformed human cell strains plus 4 Epstein-Barr virus-transformed LCLs were downloaded from the ENCODE website (http://genome.ucsc.edu/cgi-bin/hgFileUi?db=hg19&g=wgEncodeHaibMethylRrbs). There are two isogenic replicates for each cell line (except for myoblasts), which were replicates derived from the same human donor but have been treated separately, i.e., two growths of the same cell line, two separate library preparations, and two separate sequencing runs. A total of 51 DNA methylation datasets (BED files) were obtained (Supplementary Table 1).

### 4.2 Differentially methylation analysis

All statistical analyses were performed using R version 3.0.2. The identification of DMCs was performed by using the methylKit package (Akalin et al., 2012). Specifically, at each tested CpG site, we fitted a logistic regression model for the proportion of methylated cytosines in osteoblasts vs. all other samples. To be conservative, only CpGs with sequence coverage ≥ 10 × across all the cell lines were included in the analysis, and the significant DMCs were defined as CpGs showing absolute difference in methylation level of ≥ 50% between osteoblasts and other cells at a significance level of q-value ≤ 0.01. The q-values correspond to multiple testing adjusted p-values using the sliding linear model (SLIM) (Wang et al., 2011). Hierarchical clustering analysis using the significant DMCs was also carried out in the methylKit.

For the identification of DMRs, we used the eDMR package (Li et al., 2013), which can directly take objects from methylKit and perform regional optimization calling and DMR statistical analysis and filtering. Specifically, the program uses a bimodal normal distribution to identify the optimum cutoff for calling a gap between two DMRs (Li et al., 2013). The DMR identification were restricted to those regions that contain ≥ 5 CpGs including ≥ 3 DMCs and have absolute mean methylation difference > 20% between the osteoblasts and the other cell types. The statistical significance of DMRs was calculated by combining the p-values of DMCs within the refined regions through the Stouffer-Liptak test (Pedersen et al., 2012). A FDR (False Discovery Rate) correction was also applied to correct for multiple hypothesis testing for the combined p-values. The significant DMRs are those with q < 0.001.

### 4.3 Annotation analysis

DMCs and DMRs were characterized with respect to different genic regions (promoters, exons, introns, 5′UTRs, 3′UTRs and intergenic regions) and different regions relative to CGIs, including CGIs, CGI shores (2 kb regions flanking CGIs), CGI shelf (2 kb regions flanking CGI shores), and open sea (> 4 kb to the nearest CGIs). The annotation files of RefSeq genes and CGIs were downloaded from the UCSC genome browser (http://genome.ucsc.edu/cgi-bin/hgTables). In the event that a DMC was mapped to multiple different CGI regions, we assigned the DMC to a single CGI region based on the priority order: CGI > CGI shore > CGI shelf > open sea.

To assist the functional annotation of the identified DMCs/DMRs, a variety of chromatin epigenomic (histone modification marks and DNase I hypersensitivity) and transcriptomic (RNA-seq) profiles were obtained from the ENCODE project (E.P. Consortium, 2012) via the UCSC genome browser. All these transcriptomic and chromatin-related epigenomic data were generated from the same set of cell lines as those for the DNA methylation data. Additionally, we obtained combinatorial chromatin states (the 18-state model) in several cell lines (osteoblast, HSMM, GM12878, and HMEC) from the NIH Roadmap Epigenomics Consortium (Bernstein et al., 2010), which predicted the chromatin states by using the ChromHMM (Ernst and Kellis, 2012) approach on the ENCODE chromatin-related data. For the DMRs overlapping with regions of different chromatin states, we assigned them to the one state with larger proportion of overlap. Functional annotation and GO enrichment analysis of DMRs were carried out by using the GREAT package (McLean et al., 2010) with human reference genome GRCh37 (UCSC hg19, Feb/2009) as background. GREAT assigns biological meaning to a set of potential regulatory genomic regions (e.g., DMRs) by associating genomic regions with nearby genes and applying the gene annotations to the regions. Association is a two-step process. First, every gene is assigned a regulatory domain consisting of a basal domain that extends 5 kb upstream and 1 kb downstream from its TSS (regardless of other nearby genes), and an extension domain that extends in both directions up to the basal regulatory domain of the nearest upstream and downstream genes within 1 Mb (McLean et al., 2010). Then, each potential regulatory genomic region is associated with all genes whose regulatory domain it overlaps.

### 4.4 Enrichment of osteoporosis-associated genes among DMRs

We also investigated whether genes associated with DMRs are enriched for genetic variants underlying osteoporosis. Specifically, 143 genes which contain SNPs with p-value ≤ 5 × 10^− 6^ for association with bone mineral density (BMD) were recognized as osteoporosis-associated genes, based on the data released from the largest to date meta-analysis in the bone field by the by the Genetic Factors for Osteoporosis Consortium (GEFOS), including 32,961 individuals from 17 GWASs for BMD (Estrada et al., 2012). The significance of enrichment for osteoporosis-associated genes is tested by hypergeometric test comparing the DMR-annotated genes and all the 11,329 genes (as a background set) annotated to the 182,518 tested CpGs.

The following are the supplementary data related to this article.

Supplementary material Image 1

Supplementary Table 2 Full list of significant osteoblastic hyper-/hypo-methylated DMRs. Supplementary Table 2

## Disclosure of interest

All authors declare that they have no conflicts of interest.
