# Supplementary material for: Systemic analysis of osteoblast-specific DNA methylation marks reveals novel epigenetic basis of osteoblast differentiation
Source: Bone Rep. 2017 Apr 3;6:109–19. doi: 10.1016/j.bonr.2017.04.001 (PMC5384298; doi:10.1016/j.bonr.2017.04.001)
Supplement: Supplementary Table 2 — Full list of significant osteoblastic hyper-/hypo-methylated DMRs. [file mmc2.docx]

**Supplementary Table 1. Basic Characteristics of the Studied Human Cell Types**

| **Cell Type** | **Cell Line** | **Lab_ID by ENCODE** | **CpGs** | **CpGs**  **(>=10x coverage)** | **Sex & Age of Donor** | **Vendor ID of Cell Line** | **ENCODE Institutional Source** |
| --- | --- | --- | --- | --- | --- | --- | --- |
| amniotic epithelial cells | HAEpiC | SL1618 | 1112861 | 393213 | Unknown | ScienCell 7110 | UW |
| amniotic epithelial cells | HAEpiC | SL889 | 1067741 | 337118 | Unknown | ScienCell 7110 | UW |
| aortic smooth muscle cells | AoSMC | SL3546 | 1489630 | 494774 | Unknown | Lonza CC-2571 | Duke |
| aortic smooth muscle cells | AoSMC | SL766 | 1088810 | 398163 | Unknown | Lonza CC-2571 | Duke |
| Astrocytes | NH-A | SL1621 | 1137979 | 431534 | Unknown | Lonza CC-2565 | UW |
| Astrocytes | NH-A | SL887 | 1099079 | 369873 | Unknown | Lonza CC-2565 | UW |
| Astrocytes | NH-A | SL763 | 1059594 | 439762 | Unknown | Lonza CC-2565 | Duke |
| Astrocytes | NH-A | SL3544 | 1412920 | 542017 | Unknown | Lonza CC-2565 | Duke |
| cardiac fibroblasts | HCF | SL1441 | 1164037 | 397225 | Unknown | ScienCell 6300 | UW |
| cardiac fibroblasts | HCF | SL898 | 960980 | 325513 | Unknown | ScienCell 6300 | UW |
| cardiac myocytes | HCM | SL1626 | 1266647 | 487608 | Unknown | ScienCell 6200 | UW |
| cardiac myocytes | HCM | SL873 | 1351848 | 479717 | Unknown | ScienCell 6300 | UW |
| Choroid plexus epithelial cells | HCPEpiC | SL1439 | 1197499 | 404762 | Unknown | ScienCell 1310 | UW |
| Choroid plexus epithelial cells | HCPEpiC | SL892 | 1215099 | 396954 | Unknown | ScienCell 1310 | UW |
| Control myoblasts, Ctl Mb3c | HSMM Rep 2 | SL940 | 1038494 | 413461 | Female, 27 yrs | M. Ehrlich lab (Tulane) | Duke |
| Esophageal epithelial cells | HEEpiC | SL1620 | 1133015 | 413540 | Unknown | ScienCell 2720 | UW |
| Esophageal epithelial cells | HEEpiC | SL896 | 1135104 | 367303 | Unknown | ScienCell 2720 | UW |
| gum tissue fibroblasts | AG09319 | SL1418 | 1121443 | 429344 | Female, 24 yrs | Coriell AG09319 | UW |
| gum tissue fibroblasts | AG09319 | SL894 | 1084799 | 376775 | Female, 24 yrs | Coriell AG09319 | UW |
| Iris Pigment epithelial cells | HIPEpiC | SL1619 | 1114278 | 412554 | Unknown | ScienCell 6560 | UW |
| Iris Pigment epithelial cells | HIPEpiC | SL875 | 1185059 | 405376 | Unknown | ScienCell 6560 | UW |
| Lymphoblastoid cell line (LCL) | GM12891 | SL1232 | 1161222 | 431002 | Male | Coriell GM12891 | HudsonAlpha |
| LCL | GM12891 | SL1233 | 1143272 | 474650 | Male | Coriell GM12891 | HudsonAlpha |
| LCL | GM19239 | SL1804 | 1321588 | 533615 | Male | Coriell GM19939 | Duke |
| LCL | GM19239 | SL793 | 960300 | 374379 | Male | Coriell GM19239 | Duke |
| LCL | GM19240 | SL1803 | 1418190 | 615159 | Female | Coriell GM19240 | Duke |
| LCL | GM19240 | SL943 | 1198730 | 523726 | Female | Coriell GM19240 | Duke |
| LCL | GM12878 | SL727 | 1286617 | 485773 | Female | Coriell GM12878 | HudsonAlpha |
| LCL | GM12878 | SL728 | 1245151 | 474516 | Female | Coriell GM12878 | HudsonAlpha |
| Mammary epithelial cells | HMEC | SL1421 | 1137276 | 417494 | Unknown | Lonza CC-2551 | UW |
| Mammary epithelial cells | HMEC | SL867 | 1184951 | 428262 | Unknown | Lonza CC-2551 | UW |
| non-pigment ciliary epithelial cells | HNPCEpiC | SL1440 | 1189529 | 430595 | Unknown | ScienCell 6580 | UW |
| non-pigment ciliary epithelial cells | HNPCEpiC | SL877 | 1272290 | 422211 | Unknown | ScienCell 6580 | UW |
| Osteoblasts, Osteob | Osteoblasts | SL1806 | 1445851 | 582199 | Unknown | Lonza CC-2538 | Duke |
| Osteoblasts, Osteob | Osteoblasts | SL767 | 1130119 | 424869 | Unknown | Lonza CC-2538 | Duke |
| prostate epithelial cell line | PrEC | SL868 | 1292227 | 461048 | Unknown | Lonza CC-2555 | UW |
| prostate epithelial cell line | PrEC | SL869 | 1093986 | 413293 | Unknown | Lonza CC-2555 | UW |
| pulmonary alveolar epithelial cells | HPAEPiC | SL1347 | 1145206 | 398859 | Unknown | ScienCell 3200 | UW |
| pulmonary alveolar epithelial cells | HPAEPiC | SL897 | 1152966 | 386099 | Unknown | ScienCell 3200 | UW |
| Renal cortical epithelial cells | HRCEpiC | SL1344 | 1164926 | 400067 | Unknown | Lonza CC-2554 | UW |
| Renal cortical epithelial cells | HRCEpiC | SL866 | 1080077 | 358375 | Unknown | Lonza CC-2554 | UW |
| Renal epithelial cells | HRE | SL1343 | 1204514 | 429467 | Unknown | Lonza CC-2556 | UW |
| Renal epithelial cells | HRE | SL865 | 1064556 | 364127 | Unknown | Lonza CC-2556 | UW |
| renal proximal tubule epithelial cells | RPTEC | SL1346 | 1113333 | 391525 | Unknown | Lonza CC-2553 | UW |
| renal proximal tubule epithelial cells | RPTEC | SL876 | 1313940 | 476726 | Unknown | Lonza CC-2553 | UW |
| Retinal pigment epithelial cells | HRPEpiC | SL1443 | 1226178 | 448418 | Unknown | ScienCell 6540 | UW |
| Retinal pigment epithelial cells | HRPEpiC | SL874 | 1293726 | 438613 | Unknown | ScienCell 6540 | UW |
| Skin fibroblasts | Fibrobl | SL1818 | 1333723 | 562057 | Female, 10 yrs | Coriell AG08470 | Duke |
| Skin fibroblasts | Fibrobl | SL765 | 974802 | 339286 | Female, 10 yrs | Coriell AG08470 | Duke |
| Small airway epithelial cells, SA epith | SAEC | SL1342 | 1236939 | 436148 | Unknown | Lonza CC-2547 | UW |
| Small airway epithelial cells, SA epith | SAEC | SL864 | 1205009 | 404933 | Unknown | Lonza CC-2547 | UW |

Note: Samples with the same ‘Cell Line’ ID are isogenic replications, which are technical replicates from biosamples derived from the same human donor. These biosamples have been treated separately, i.e., two growths of the same cell line, two separate library preparations, and two separate sequencing runs.

**Supplementary Table 2. Full List of Significant Osteoblastic Hyper-/Hypo-Methylated DMRs**

Please see attached Excel file “Supplementary Table 2_Full List of Significant DMRs.xlsx”.

**Supplementary Table 3. Results of DMR Analysis for Known Primary Osteoblastic Genes**

| **Gene** | **Gene Position** | **DMRs** | **Num.CpGs** | **Num.DMCs** | **DMR.qvalue** | **Mean.DM%** |
| --- | --- | --- | --- | --- | --- | --- |
| Runx2 | chr6:45296054-45339790 | NA | NA | NA | NA | NA |
| Sp7 | chr12:53720360-53738577 | chr12:53738647-53738719 | 3 | 3 | 0.25 | -1.36 |
| Dlx5 | chr7:96649702-96654143 | chr7:96645870-96645965 | 5 | 5 | 0.02 | -3.20 |
| Msx2 | chr5:174151575-174157902 | chr5:174151905-174151937 | 4 | 4 | 0.07 | -0.88 |
| **BGLAP** | **chr1:156211753-156213123** | **chr1:156211407-156211474** | **5** | **5** | **7.15E-45** | **-44.89** |
| COL1A1 | chr17:48261457-48279000 | NA | NA | NA | NA | NA |
| MEF2C | chr5:88014058-88199922 | NA | NA | NA | NA | NA |
| BMP2 | chr20:6748745-6760925 | chr20:6750828-6750834 | 3 | 3 | 0.14 | -1.99 |
| BMP4 | chr14:54416455-54423609 | chr14:54423395-54423484 | 3 | 3 | 0.12 | -0.51 |
| BMP6 | chr6:7726332-7881961 | NA | NA | NA | NA | NA |
| BMP7 | chr20:55743809-55841707 | chr20:55754610-55754635 | 5 | 5 | 6.79E-05 | 13.27 |
| BMP9 | chr10:48411774-48416872 | NA | NA | NA | NA | NA |
| WNT6 | chr2:219724523-219738577 | chr2:219724352-219724460 | 3 | 3 | 0.03 | 2.70 |
|  |  | chr2:219738308-219738337 | 6 | 6 | 0.004 | -5.30 |
| WNT8 | chr5:137419581-137427230 | NA | NA | NA | NA | NA |
| WNT10a | chr2:219745255-219758651 | chr2:219745568-219745643 | 5 | 5 | 0.04 | -0.69 |
| WNT10b | chr12:49359123-49365641 | chr12:49359976-49360052 | 3 | 3 | 0.002 | -0.71 |

Note: Results for all analyzed potential DMRs within 5kb upstream and 5kb downstream regions of the selected genes are presented. “NA” means that no qualified (sequence coverage ≥10x) CpGs were mapped to the corresponding regions.

**Supplementary Figure 1. Distribution of DMCs across the genome.**

**Supplementary Figure 2. Hierarchical cluster analysis using the significant DMCs.**

**
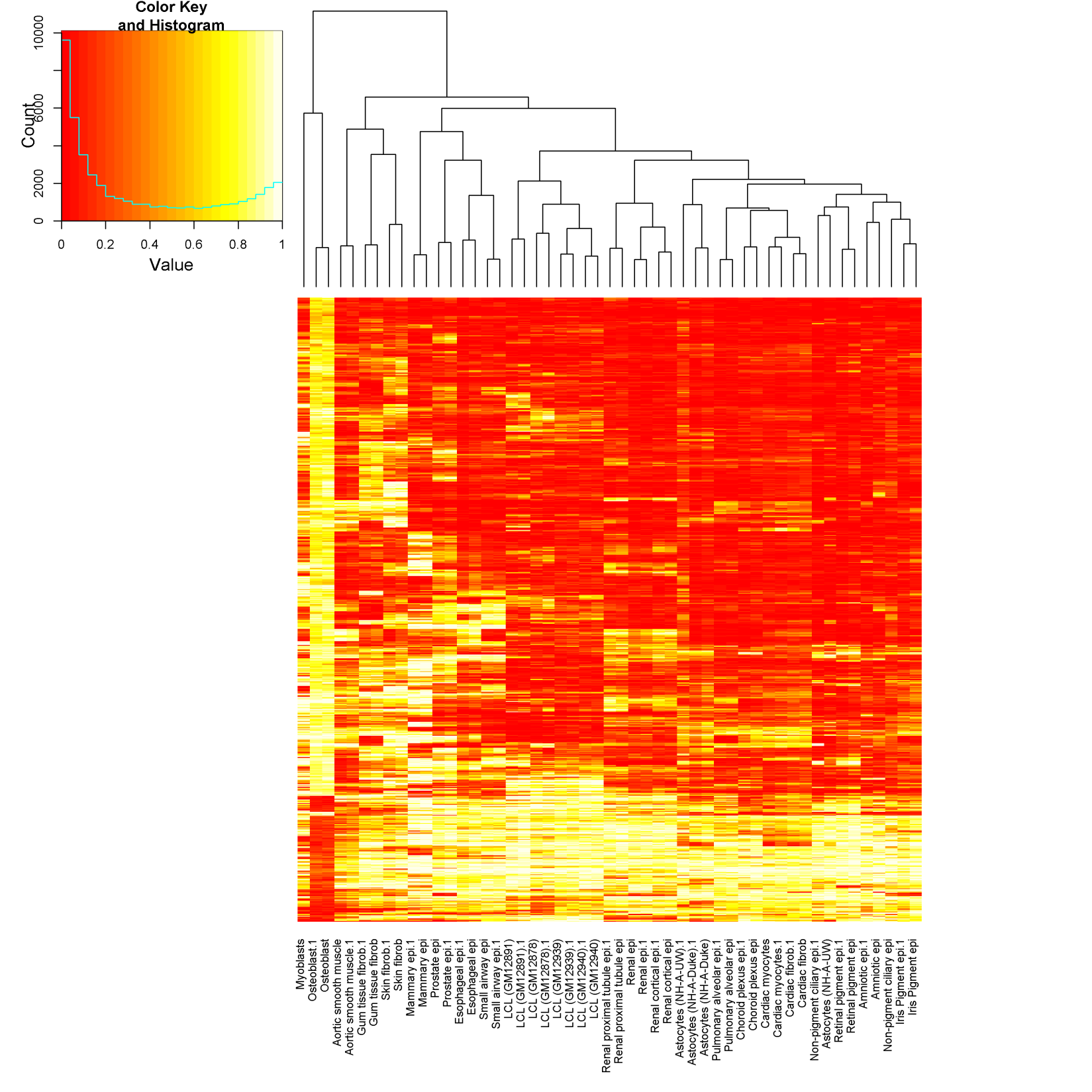
**

The heatmap shows the alternation of the methylation status, with yellow and red indicating high and low DNA methylation levels, respectively.

**Supplementary Figure 3.** Distribution of the osteoblast-specific hyper- and hypo-methylated DMRs across (A) different regions related to CGIs, (B) different genic regions, (C) regions of different distance from the nearest TSSs as determined by GREAT analysis [[32](#_ENREF_32)] , and (D) different types of chromatin segments in osteoblasts.


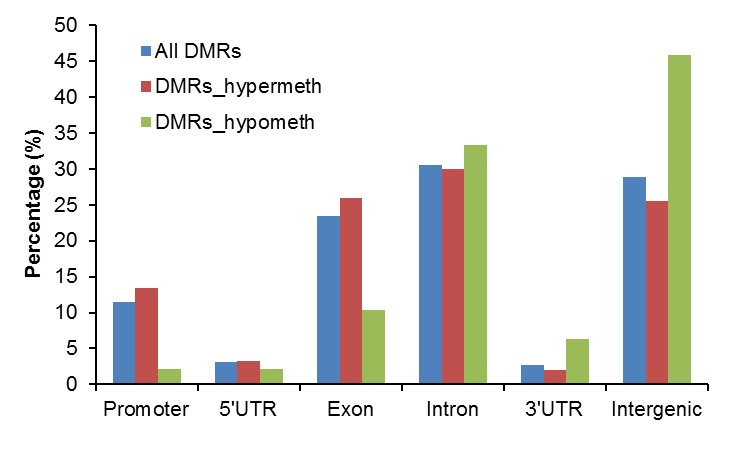

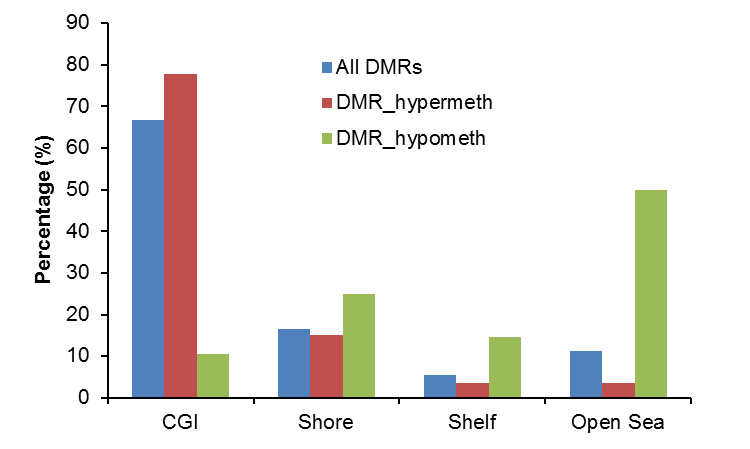


**B.**

**A.**

**D.**

**C.**


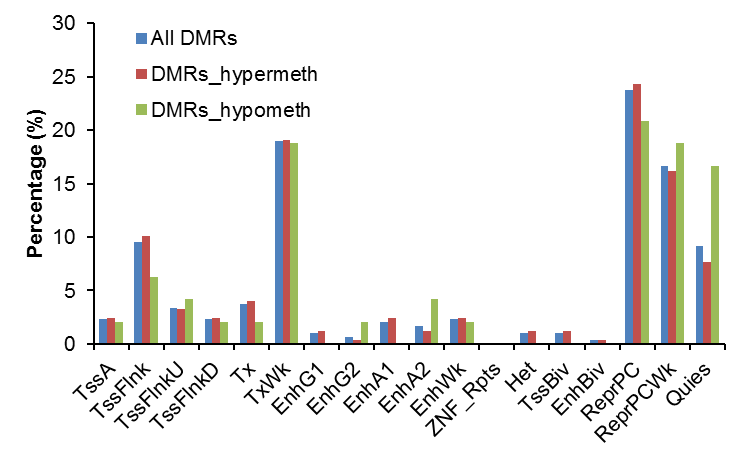

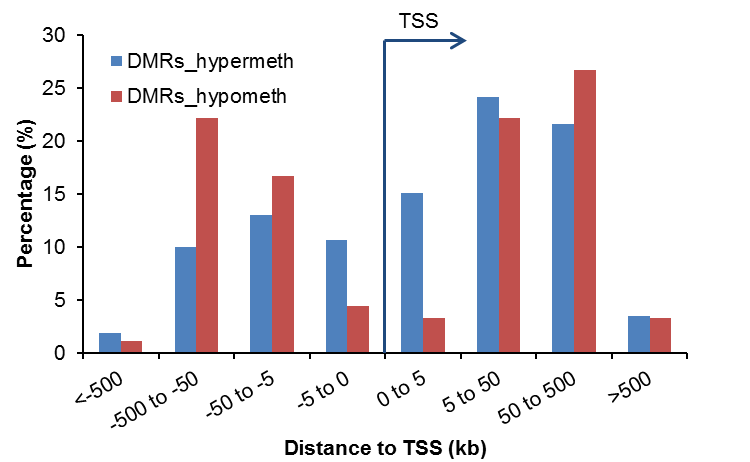


The 18 chromatin states were determined by NIH Roadmap Epigenome project based on ChIP-seq data of histone modification marks (H3K4me3, H3K4me1, H3K36me3, H3K27me3, H3K9me3, H3K27ac) across 98 epigenomes. TssA, Active TSS; TssFlnk, Flanking TSS; TssFlnkU, Flanking TSS Upstream; TssFlnkD, Flanking TSS Downstream; Tx, Strong transcription; TxWk, Weak transcription; EnhG1, Genic enhancer1; EnhG2, Genic enhancer2; EnhA1, Active Enhancer 1; EnhA2, Active Enhancer 2; EnhWk, Weak Enhancer; ZNF/Rpts, ZNF genes & repeats; Het, Heterochromatin; TssBiv, Bivalent/Poised TSS; EnhBiv, Bivalent Enhancer; ReprPC, Repressed PolyComb; ReprPC, Weak Repressed PolyComb; Quies, Quiescent/Low.

**Supplementary Figure 4. Osteoblastic hypermethylation at *SIM2* TSS surrounding region.**

The following profiles are shown using the UCSC Genome Browser (<http://genome.ucsc.edu>, version hg19) for the *SIM2* gene region: UCSC genes, RefSeq genes, RNA-seq data from ENCODE/Cold Spring Harbor, DNA methylation signal assessed by RRBS from ENCODE/HudsonAlpha; histone modification data (H3K4me1, H3K4me3, H3K9me3, H3K27ac, H3K27me3, H3K36me3, H3K79me2, and H4K20me1) by ChIP-seq from ENCODE/Broad, and CpG islands. LCL (GM12878), HMEC, HSMM, and osteoblast (HOB) are the only studied cell types having both data in RNA-seq and histone modification tracks available from ENCODE. Osteoblast-specific DMRs are indicated in the blue box. For the histone modification data, the shading of the bars is proportional to the intensity of the signal.

**Supplementary Figure 5. Osteoblastic hypermethylation at *GLIS1* TSS surrounding region.**

The same tracks as illustrated in Supplementary Figure 4 are shown using the UCSC Genome Browser (<http://genome.ucsc.edu>, version hg19). Osteoblast-specific DMRs and its associated histone modification marks are highlighted in the blue box.

**Supplementary Figure 6. Osteoblastic hypermethylation at *MEST* gene region.**

The same tracks as illustrated in Supplementary Figure 4 are shown using the UCSC Genome Browser (<http://genome.ucsc.edu>, version hg19), with the additions of RNA-seq tracks for LCL (GM12891 and GM12892) from ENCODE/Caltech. Osteoblast-specific DMR and its associated histone modification marks are highlighted in the blue box.

**Supplementary Figure 7. Various isoforms and osteoblastic hypomethylation at *NRXN2* gene region.**

**A.**

**B.**

(A) *NRXN2* isoforms annotated by NCBI Homo sapiens Annotation Release 107. The specific isoform corresponds to the mRNA transcript detected in osteoblast are highlighted in red box. (B) The same tracks as illustrated in Supplementary Figure 4 are shown using the UCSC Genome Browser (<http://genome.ucsc.edu>, version hg19). Osteoblast-specific DMR and its associated histone modification marks are highlighted in the blue box.

**Supplementary Figure 8. Osteoblastic hypomethylation at *BGLAP* gene region.**

The same tracks as illustrated in Supplementary Figure 4 are shown using the UCSC Genome Browser (<http://genome.ucsc.edu>, version hg19). Osteoblast-specific DMR and its associated histone modification marks are highlighted in the blue box.
